# Presence of tophi is associated with a rapid decline in the renal function in patients with gout

**DOI:** 10.1038/s41598-021-84980-6

**Published:** 2021-03-11

**Authors:** Yoon-Jeong Oh, Ki Won Moon

**Affiliations:** Division of Rheumatology, Department of Internal Medicine, Kangwon National University School of Medicine, 156, Baengnyeong-ro, Chuncheon-si, Gangwon-do Republic of Korea

**Keywords:** Immunology, Nephrology, Rheumatology

## Abstract

We aimed to compare clinical characteristics of patients with and without tophi at the time of the diagnosis of gout and investigate the association of tophi and renal function in gout patients. The patients who were first diagnosed with gout at the Kangwon National University Hospital were retrospectively studied. Patients were divided into 2 groups according to the presence of tophi at the diagnosis. We compared clinical characteristics and the progression of renal dysfunction between the two groups. Of 276 patients, 66 (25.5%) initially presented with tophi. Tophi group was older, had a longer symptom duration, and a higher prevalence of multiple joint involvement than those without tophi. In multivariate logistic regression analysis, prolonged symptom duration and multiple joint involvement were significantly associated with increased risk of formation of tophi. The decline in the eGFR was more prominent in patients with tophi than in those without (− 4.8 ± 14.5 vs. − 0.7 ± 11.9 ml/min/1.73 m^2^/year, respectively; *P* = 0.039). The presence of tophi was significantly associated with a rapid decline in the eGFR (β = − 0.136; *P* = 0.042). In conclusion, the presence of tophi was associated with a rapid declining renal function. Therefore, an early diagnosis and closely monitoring of renal function might be important in gout patients with tophi.

## Introduction

Uric acid primarily exists as soluble monosodium urate (MSU) under physiological conditions in humans. However, when the urate concentration exceeds the limit of solubility, it increases the risk of MSU crystal formation and precipitation^1^. Hyperuricemia results in serious complications including gout, tophi, kidney stones, urate nephropathy, and the subsequent loss of kidney function^2^. Of these, gout is the most common inflammatory arthritis resulting from a chronic deposition of MSU crystals in the joints and other soft tissues^3^.

Tophi is primarily formed due to an inflammatory response to MSU crystals in which inflammatory cells infiltrate the MSU crystals around the joints. This usually occur in longstanding, undiagnosed, or improperly controlled cases of gout, though in rare cases, they may present as an early-stage symptom of gout^4,5^. Intra-articular tophi may sometimes result in bone destruction, joint deformities, and dysfunction, and muscle weakness which can adversely affect the patient’s quality of life^6–8^. Furthermore, a recent report showed that the presence of tophi was also associated with an increased risk of mortality in gout patients^9^.

It has been reported that several studies about mutual relationship between gout and its associated comorbidities. A previous study reported that patients with tophaceous gout show an increased risk of cardiovascular diseases (CVD)^10^. It is well known that hyperuricemia is potentially associated with the development and progression of comorbidities, including CVD, metabolic syndrome, and renal disease^11^. Recently, the prevalence of comorbidities among gout patients has been also increasing with the rapid rise in the number of gout patients^12^. In addition, several comorbidities including heart failure (HF), hypertension (HTN), and diabetes mellitus (DM) may play a dominant role in MSU crystal deposition in these patients^13^.

Lu et al. reported that patient with early-onset tophaceous gout are more likely to develop renal dysfunction^14^, however, few studies have investigated if the presence of tophi is related with the progression of renal dysfunction in gout patients. Therefore. this study aimed to compare the clinical characteristics of patients with and without tophi at the time of diagnosis of gout and investigate the association of tophi and renal function in gout patients.

## Results

### Baseline characteristics of gout patients with and without tophi

From a total of 257 patients with gout, 66 (25.7%) had tophi whereas the remaining 191 (74.3%) had no tophi at the time of the initial visit. The baseline characteristics of the study patients with and without tophi are shown in Table 1. The mean age of patients in the tophi group at diagnosis was significantly higher than that of patients in the no tophi group (61.0 ± 15.9 years vs. 52. 0 ± 17.2 years, respectively; *P* < 0.001). The gender distribution was similar between the 2 groups. The median duration of symptoms prior to the diagnosis of gout was significantly longer in the tophi group than in the no tophi group (63.7 [11.0–147.0] months vs. 17.9 [2.6–56.3] months, respectively; *P* < 0.001). The 2 groups did not differ with respect to factors such as a history of renal stones, the family history, number of gout flares (≥ 3 per year), concomitant medication (colchicine, diuretics, and aspirin use), type of urate lowering therapy (ULT), presence of comorbidities (HTN, DM, CVD, HF, dyslipidemia, hypertriglyceridemia, chronic kidney disease [CKD], dementia, and past history of cancer). Among the study subjects, 162 (63.0%) patients were initially treated with febuxostat and 95 (37.0%) patients were treated with allopurinol. In the febuxostat group, 160/162 (98.8%) patients continued taking febuxostat during the follow-up period, and 2 patients discontinued the drug. In the allopurinol group, 84 patients changed to febuxostat and 11 patients stopped taking allopurinol. At baseline, the uric acid, blood urea nitrogen (BUN), creatinine (Cr) and estimated glomerular filtration rate (eGFR) values, too, did not differ between the 2 groups.

**Table 1:** Baseline characteristics according to the presence of tophi.

Variables	Tophi (N = 66)	No tophi (N = 191)	*P* value
Age at diagnosis (years)	61.0 ± 15.9	52.0 ± 17.2	< 0.001
Age at symptom onset (years)	54.8 ± 17.0	49.8 ± 17.3	0.051
Gender (male)	58 (87.9)	179 (93.7)	0.179
Symptom duration (months)	63.7 (11.0–147.0)	17.9 (2.6–56.3)	< 0.001
Renal stone	6/58 (10.3)	18/150 (12.0)	0.814
Family history	3 (4.5)	11 (5.8)	1.0
Gout flares (≥ 3 per year)	6 (9.1)	9 (4.7)	0.223
**Type of ULT**			0.185
Febuxostat	37 (56.1)	125 (65.4)	
Allopurinol	29 (43.9)	66 (34.6)	
Colchicine use	53 (80.3)	151 (79.1)	1.0
Diuretics use	9 (13.6)	28 (14.7)	0.843
Aspirin use	10 (15.2)	37 (19.4)	0.580
**Comorbidities**			
Hypertension	38 (57.6)	86 (45.0)	0.088
Diabetes mellitus	9 (13.6)	34 (17.8)	0.566
Cardiovascular disease	16 (24.2)	41 (21.5)	0.731
Heart failure	2 (3.0)	9 (4.7)	0.734
Dyslipidemia	20 (30.3)	65 (34.0)	0.650
Hypertriglyceridemia	25 (49.0)	74 (48.7)	1.0
Chronic kidney disease (eGFR < 60 ml/min/1.73 m^2)^	17 (25.8)	46 (24.1)	0.868
Dementia	1 (1.5)	3 (1.6)	1.0
Past history of cancer	3 (4.5)	18 (9.4)	0.299
Charlson comorbidity index	2.9 ± 2.3	2.5 ± 2.8	0.292
**Laboratory findings**			
Uric acid (mg/dL)	8.8 ± 2.2	9.0 ± 1.8	0.487
BUN (mg/dL)	21.0 ± 9.7	18.4 ± 10.1	0.067
Cr (mg/dL)	1.1 ± 0.4	1.2 ± 0.5	0.782
eGFR (ml/min/1.73m^2^)	76.2 ± 26.0	81.6 ± 28.5	0.164
Total cholesterol (mg/dL)	174.3 ± 42.0	180.9 ± 47.3	0.306
Triglyceride (mg/dL)	229.8 ± 177.3	244.6 ± 170.6	0.622
LDL-C (mg/dL)	103.4 ± 33.7	107.4 ± 38.7	0.504
HDL-C (mg/dL)	44.2 ± 14.1	44.2 ± 11.7	0.979

Results are expressed as the mean ± SD, as the median (interquartile range, IQR), or as number (%).

*ULT* urate lowering therapy, *BUN* blood urea nitrogen, *Cr* creatinine, *eGFR* estimated glomerular filtration rate, *LDL-C* low-density lipoprotein cholesterol, *HDL-C* high-density lipoprotein cholesterol.

### Characteristics of joint involvement in patients with and without tophi

Table 2 shows a comparison of the characteristics of the affected joints in patients with and without tophi. The mean number of tophi in patients of the tophi group was 2.3 ± 1.9. At baseline, the number of affected joints of patients in the tophi group was much higher than that of patients in the no tophi group (3.0 ± 2.1 vs. 1.4 ± 0.6, respectively; *P* < 0.001). The involvement of the upper extremities was much more prevalent in patients of the tophi group than in patients of the no tophi group (36.4% *vs*. 7.3%, respectively; *P* < 0.001). In contrast, the involvement of the lower extremities was much less prevalent in the patients of the tophi group than in patients of the no tophi group (89.4% vs. 99.5%, respectively; *P* < 0.001). The involvement of both upper and lower extremities was much higher in the patients of the tophi group than in patients of the no tophi group (22.7% vs. 6.8%, respectively; *P* = 0.001). Solitary involvement of the small joints was more frequent in the patients of the no tophi group than in patients of the tophi group (59.7% *vs*. 42.4%, respectively; *P* = 0.021).

**Table 2:** Involved joints in patients with gout at baseline.

Variables	Tophi (N = 66)	No tophi (N = 191)	*P* value
Mean tophi number	2.3 ± 1.9		
Number of involved joints	3.0 ± 2.1	1.4 ± 0.6	< 0.001
Involvement of only large joints	12 (18.2)	46 (24.1)	0.394
Involvement of only small joints	28 (42.4)	114 (59.7)	0.021
Involvement of multiple joints	34 (51.5)	18 (9.4)	< 0.001
Involvement of upper extremities	24 (36.4)	14 (7.3)	< 0.001
Involvement of lower extremities	59 (89.4)	190 (99.5)	< 0.001
Involvement of upper and lower extremities	15 (22.7)	13 (6.8)	0.001

Results are expressed as the mean ± SD, or as number (%).

### Risk factors associated with the development of tophi at the time of diagnosis of gout

Multivariate logistic regression analysis was performed to evaluate the risk factors associated with the development of tophi at the time of diagnosis of gout in the study patients (Table 3). After adjusting for the clinical variables, a prolonged duration of symptoms (Odds ratio [OR], 1.010; 95% confidence interval [CI], 1.004–1.017; *P* = 0.001) and a high number of involved joints (OR, 3.027; 95% CI, 1.831–5.004; *P* < 0.001) were found to be independent risk factors for the presence of tophi at the initial diagnosis of gout in this study.

**Table 3:** Logistic regression analysis for the presence of tophi in study subjects.

Baseline variable	Univariate		Multivariate^a^	
	OR (95% CI)	*P*	OR (95% CI)	*P*
Age at diagnosis (years)	1.033 (1.015–1.051)	< 0.001	1.023 (0.997–1.050)	0.088
Gender (male)	2.057 (0.802–5.279)	0.133		
Symptom duration (months)	1.014 (1.009–1.019)	< 0.001	1.010 (1.004–1.017)	0.001
Number of involved joints	3.871 (2.574–5.821)	< 0.001	3.027 (1.831–5.004)	< 0.001
Involved of large joint	2.428 (1.366–4.316)	0.003	0.796 (0.350–1.809)	0.586
Involved of small joint	1.293 (0.648–2.582)	0.466		
Involve of upper extremities	7.224 (3.447–15.142)	< 0.001	2.095 (0.768–5.719)	0.149
HTN	1.657 (0.941–2.916)	0.080	1.077 (0.491–2.360)	0.854
CVD	1.171 (0.605–2.266)	0.640		
CKD	1.094 (0.575–2.082)	0.785		
Baseline uric acid (mg/dL)	0.940 (0.804–1.099)	0.437		

*OR* odds ratio, *CI* confidence interval, *HTN* hypertension, *CVD* cerebrovascular disease, *CKD* chronic kidney disease.

^a^Adjusted for age at diagnosis, symptom duration, number of involved joints, involved of large joint, involve of upper extremities, and HTN.

### Follow-up data in study subjects

**Figure 1:**
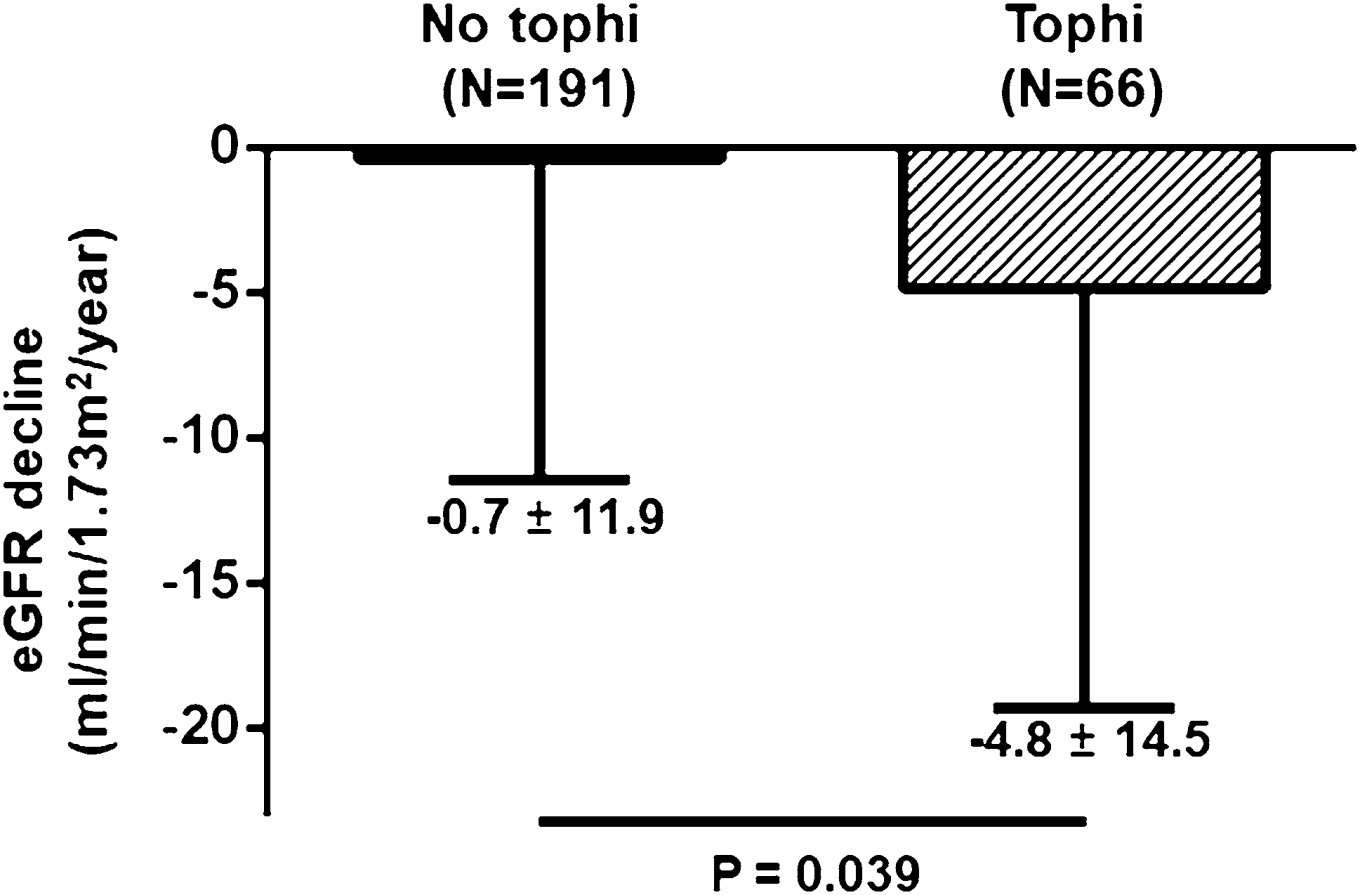
Changes in eGFR in gout patients with or without tophi. Decline of the eGFR in the tophi group was − 4.8 ± 14.5 ml/min/1.73m^2^/year, while that in the no tophi group was − 0.7 ± 11.9 ml/min/1.73m^2^/year (*P* = 0.039).

The median duration of follow-up was significantly longer in the tophi group than in the no tophi group (61.9 [22.6–91.6] months *vs*. 34.9 [18.7–66.4] months, *P* = 0.004) (Table 4). Out of 257 patients, 244 patients maintained the ULT during the follow-up period, and 13 patients discontinued it. Patients that received non-steroidal anti-inflammatory drugs (NSAIDs) were 43 (65.2%) in patients with tophi and 127 (66.5%) in patients without tophi (*P* = 0.881). There was no significant difference in NSAIDs consumption days between the two groups (34.9 ± 76.0 days/year vs. 24.7 ± 88.5 days/year, *P* = 0.404). Moreover, there was no significant difference in the mean uric acid level during follow-up between the two groups (5.3 ± 1.4 in the tophi group vs. 5.4 ± 1.2 in the no tophi group, *P* = 0.619). There was also no significant difference in the number of patients who reached the treatment target of more than 80% between the two groups (34 [51.5%] in the tophi group *vs*. 93 [48.7%] in the no tophi group, *P* = 0.775). The rate of decline in the eGFR was significantly higher in the tophi group than in the no tophi group (− 4.8 ± 14.5 ml/min/1.73m^2^/year vs. − 0.7 ± 11.9 ml/min/1.73m^2^/year, respectively; *P* = 0.039) (Fig. 1).

**Table 4:** Changes in the uric acid, eGFR decline, and medications during the follow-up period.

Variables	Tophi (N = 66)	No tophi (N = 191)	*P* value
Follow-up duration (months)	61.9 (22.6–91.6)	34.9 (18.7–66.4)	0.004
Mean uric acid (mg/dL)	5.3 ± 1.4	5.4 ± 1.2	0.619
Maintenance of ULT	64 (97.0)	180 (94.2)	0.525
Response of ULT	34 (51.5)	93 (48.7)	0.775
NSAID use	43 (65.2)	127 (66.5)	0.881
NSAID consumption (days/year)	34.9 ± 76.0	24.7 ± 88.5	0.404
eGFR decline rate (ml/min/1.73m^2^/year)	− 4.8 ± 14.5	− 0.7 ± 11.9	0.039

Results are expressed as the mean ± SD, or as number (%).

*ULT* urate lowering therapy, *NSAID* non-steroidal anti-inflammatory drugs, *eGFR* estimated glomerular filtration rate.

### Evaluation of the presence of tophi as an independent risk factor for a rapid decline in the eGFR in gout patients

The risk factors associated with a rapid decline in the eGFR were analyzed using linear regression analysis (Table 5). After adjusting for symptom duration, eGFR at baseline, presence of tophi, use of NSAIDs, type of ULT, and mean uric acid levels, the eGFR at baseline (β = − 0.247; *P* < 0.001) and the presence of tophi at diagnosis (β = − 0.136; *P* = 0.042) were significantly associated with a rapid decline in the eGFR in study subjects.

**Table 5:** Linear regression analysis for eGFR decline in study patients.

Baseline variable	Univariate		Multivariate^a^	
	β	*P*	β	*P*
Age at diagnosis (years)	− 0.048	0.441		
Gender (male)	− 0.066	0.295		
Symptom duration (months)	− 0.122	0.05	− 0.045	0.504
Number of involved joints	− 0.032	0.611		
Use of NSAIDs	− 0.017	0.780	0.044	0.490
Type of ULT	− 0.070	0.260	− 0.079	0.198
Mean uric acid (mg/dL)	0.030	0.636	0.089	0.164
eGFR (ml/min/1.73m^2^/year)	− 0.207	0.001	− 0.247	< 0.001
Presence of tophi	− 0.143	0.022	− 0.136	0.042

*NSAID* non-steroidal anti-inflammatory drugs, *ULT* urate lowering therapy, *eGFR* estimated glomerular filtration rate.

^a^Adjusted for symptom duration, use of NSAID, type of ULT, mean uric acid levels, baseline eGFR and tophi.

## Discussion

This study demonstrated that a prolonged duration of symptoms and a high number of involved joints were significantly associated with the presence of tophi at the time of diagnosis in gout patients. In addition, we showed that the presence of tophi at diagnosis was significantly associated with a declining renal function.

A suboptimal treatment of gout contributes to increased frequency of gout attacks leading to chronic tophi formation and joint deformities. It has been reported that the prevalence of tophi in patients with gout varies from 3 to 21%^15^. During a gout attack, the MSU crystals activate monocytes and macrophages with the production of chemotactic substances causing a massive infiltration of neutrophils in the joint fluid and synovial membrane. Simultaneously, these crystals induce the secretion of a variety of cytokines, prostanoids, chemotactic factors, and other proteins which amplify the inflammatory process through the recruitment of inflammatory cells, the upregulation of adhesion molecules, and the stimulation of the acute phase response^16,17^. Therefore, the stimulation of immune and inflammatory cells by the MSU crystals may lead to acute pain if not properly resolved, resulting in tophi formation and joint damage in the form of bone erosions and joint dysfunction. The objective of long-term management of gout is to maintain low levels of serum uric acid and prevent chronic complications such as tophi formation and chronic damage of the affected joints.

Interestingly, joint involvements of upper extremities were more observed in the tophi group compared to no tophi group in this study. Gout attack occurs predominantly in the first metatarsophalangeal joint (1st MTP joint), and many as 50–70% of first gout attacks occurs at this joint^18^. Our study also showed that most patients had symptoms in the 1st MTP joint of the lower extremities. However, since the duration of symptom was much longer in the tophi group, several joints would be inevitably affected and the involvement of the upper extremities was more frequently observed in the tophi group than in the no tophi group (36.4% vs. 7.3%, *P* < 0.001) (Table 2). A previous study reported that the duration of the disease and number of involved joints were associated with the development of tophi in gout patients^19^. The current study also demonstrated that a prolonged duration of symptoms without treatment and a high number of affected joints were significantly associated with tophi formation (Table 3). In terms of uncontrolled hyperuricemia, a long period of chronic gouty symptoms was closely associated with the occurrence of tophaceous nodules in gout patients.

Previous studies have shown that multiple factors including an advanced age or a reduced creatinine clearance at baseline are associated with the early formation of tophi^19,20^. Gancheva et al. reported that a reduced creatinine clearance was an independent risk factor for tophaceous gout^21^. Lu et al. also showed that pre-existing tophi play an important role in decreasing the eGFR in early-onset juvenile tophaceous gout patients, whose mean age of onset of gout was 15.7 years for tophi patients and 16.8 years for non-tophi patients^14^. The current study, in which the mean age of onset of gout was 51.1 years, also showed that the presence of tophi at diagnosis was associated with a progressive decline in renal function even though there was no difference in the baseline renal function between the groups with and those without tophi. Previous studies have reported that the prevalence of gout has increased in recent decades, and there is a shift in the age of onset of gout to a much younger age group than before because of an increased prevalence of metabolic syndrome, obesity, and from diet-related causes^22^. Along with metabolic syndrome, the prevalence of CKD is also increasing in these populations together with an increased prevalence of CVD^23^. In addition, increased MSU crystal deposition is associated with an increased risk of urate renal stone formation. Therefore, tophi, as markers of long-standing gout, are associated with an increased risk of progression of CKD. Several pathophysiological associations between the presence of tophi and a decline in renal function can be explained. NSAIDs are nephrotoxicitic agents, and long-term use of NSAIDs is associated with an increased risk of NSAIDs-related nephrotoxicity^24^. It is a possible that a patient with tophi might be frequently exposed to analgesics including NSAIDs because they suffer from inflammation and its related symptoms. However, there was no difference in the number of NSAIDs consumption days between the two groups in our study. There is another possibility that it is related to urate nephropathy. A previous study by Ayoub et al., reported that biopsy-proven medullary tophi may contribute the progression of CKD^25^. Medullary tophi may destroy and block the collecting system in the kidneys and induce CKD. This pathologic abnormality might be prevalent in the tophaceous gout patients. However, most of the patients in this study did not undergo renal image, so further studies are needed.

There are several limitations to this study. First, the present study has a retrospective cohort design and the study population was constituted by patients registered at a single medical center. Therefore, the size of the study sample was relatively small. Second, as this study was based on data derived from electronic medical records, it lacks detailed further follow-up data, pre-existing tophi may have remained undetected by both the individual as well as the healthcare professionals. Third, as the diagnosis of few of the tophi was not confirmed via microscopy, on ultrasound, or using dual energy computed tomography, they could not be distinguished from other similar diseases characterized by crystal deposition including the deposition of calcium pyrophosphate crystals. Finally, since the duration of symptoms was longer in the tophi group, we are not able to exclude long-standing, untreated patients with gout in this group. Subgroup analysis was performed according to symptom duration of gout. Out of 101 patients with symptom durations of < 1 year, 18 (17.8%) patients had tophi. Meanwhile, 48/156 (30.8%) patients with symptom durations of ≥ 1 year were in the tophi group. In the subgroup with a duration of symptoms of < 1 year, the rate of decline in eGFR was − 2.9 ± 5.7 ml/min/1.73m^2^ /year in patients with tophi and 0.1 ± 15.4 ml/min/1.73m^2^/year in patients without tophi (*P* = 0.421). In patients with symptom duration of ≥ 1 year, the decline in eGFR was − 5.6 ± 16.6 ml/min/1.73 m^2^/year in the tophi group and − 1.3 ± 8.3 ml/min/1.73m^2^/year in the no tophi group (*P* = 0.097). Regardless of the duration of symptom, we found that the eGFR of patients with tophi tended to decrease more rapidly than those without tophi, but this was not statistically significant even though it might be because the number of subgroup subjects was relatively small. Therefore, further large-scaled study is needed to confirm our results. However, the strength of the present study is that 2 groups were relatively well balanced without significant differences in the renal function, uric acid levels, and use of diuretics at the time of diagnosis of gout. This made it easier to identify the risk factors responsible for the progression of renal dysfunction in gout patients.

In conclusion, our results suggest that a prolonged duration of symptom prior to the diagnosis of gout and a high number of involved joint may contribute to the presence of tophi as the initial manifestation at diagnosis. In addition, the presence of tophi at the time of diagnosis of gout was significantly associated with a rapid decline in the renal function. Therefore, early diagnosis and appropriate treatment are essential to prevent a further deterioration of the kidney function in tophaceous gout patients.

## Methods

A total of 257 patients who were first diagnosed with gout at the Kangwon National University Hospital (KNUH) from January 2012 to December 2018 were included in this study. All study subjects were prescribed febuxostat or allopurinol as ULT and maintained it for more than 3 months. The following patients were excluded: age at the time of gout diagnosis < 18 years, patients who were treated with ULT for asymptomatic hyperuricemia, patients on dialysis, and those with a follow-up period of less than 3 months. Gout was diagnosed according to the 2015 American College of Rheumatology/European League Against Rheumatism Gout Classification Criteria^26^. The frequency of the gout flares and the date of the initial symptoms were self-reported by the patients. The duration of symptoms was defined as the duration between the first clinical manifestation of gout and the initiation of ULT. The presence and number of tophi were determined by experienced rheumatologists during the initial assessment. Most patients with gout that have tophi were found to have monosodium urate crystal using polarized light microscopy by arthrocentesis, and some of those were diagnosed using imaging modalities, such as ultrasonography or dual energy computerized tomography, and others were clinically confirmed by physical examination without joint fluid analysis. Tophi were classified according to the size (small or large) and location of the affected joints (upper extremities [elbow, wrist, metacarpophalangeal joints, proximal phalangeal joints, and distal phalangeal joints] or lower extremities [knee, ankle, and metatarsophalangeal joints]). The study was approved by the Institutional Review Board of Kangwon National University Hospital and conducted in accordance with the Declaration of Helsinki (KNUH IRB protocol number: 2019-12-009). Informed consent was waived by the KNUH institutional review board due to the retrospective nature of this study.

### Data collection

All data were retrieved from the electronic medical records of KNUH. Demographic data, including the age, gender, family history, history of renal stones, frequency of gout flares, involved joints, concomitant medications (ULT, NSAIDs, colchicine, aspirin, diuretics including furosemide and thiazide), and data on comorbidities such as HTN, DM, CVD, HF, dyslipidemia, hypertriglyceridemia, CKD, dementia, and a past history of cancer were gathered. We also collected the following biochemical laboratory data: serum uric acid, BUN, Cr, eGFR, total cholesterol, triglyceride, low-density lipoprotein cholesterol and high-density lipoprotein cholesterol levels at the time of diagnosis. The uric acid levels at each outpatient visit were obtained. In addition, the levels of BUN, Cr, and eGFR at the last follow-up were also obtained. To find out the response to ULT, whether the uric acid level reached the ≤ 6.0 mg/dL was assessed at each outpatient visit. We defined a patient as the response group when their uric acid levels reached ≤ 6.0 mg/dL at 80% or more of total number of visits. The change in the eGFR level from that at the time of diagnosis of gout to that at the last follow-up date was calculated. The eGFR was determined using the Modified of Diet in Renal Disease formula^27^.

### Statistical analysis

Continuous variable are expressed as the mean ± standard deviation (SD) or as the median (interquartile range, IQR), while categorical variables are expressed as number percentages (%). The Chi-square test was used to compare the categorical variables between patients with and without tophi. Continuous variables were compared using Student’s *t*-test for parametric data or the Mann–Whitney U test for nonparametric data. Multivariate logistic regression analysis was performed to estimate the relative risk of tophi formation in the study subjects. Variables that had a *P* value of < 0.1 on univariate analysis were selected for multivariate analysis. In addition, factors associated with a decline in the eGFR were identified using the linear regression model. The clinical variables that had a *P* value of < 0.1 on univariate analysis, use of NSAIDs, type of ULT, and mean uric acid levels were used for multivariate analysis. All statistical analyses were performed using SPSS (version 23.0, Chicago, IL). A *P *value < 0.05 was considered statistically significant.
